# Natural Products for Cancer Prevention and Interception: Preclinical and Clinical Studies and Funding Opportunities

**DOI:** 10.3390/ph17010136

**Published:** 2024-01-20

**Authors:** Edward R. Sauter, Altaf Mohammed

**Affiliations:** 1 Breast and Gynecologic Cancer Research Group, Division of Cancer Prevention, National Cancer Institute, Rockville, MD 20850, USA; 2 Chemopreventive Agent Development Research Group, Division of Cancer Prevention, National Cancer Institute, Rockville, MD 20850, USA; altaf.mohammed@nih.gov

**Keywords:** cancer prevention, natural products, cancer interception, combination chemoprevention, natural products, cancer prevention, cancer interception

## Abstract

Multiple agents derived from natural products (NPs) have been evaluated for cancer prevention and interception, either alone or in combination. The National Cancer Institute (NCI) is very interested in advancing research to identify additional agents that, alone or in combination, may prove useful in cancer prevention. Below, we provide an overview of NP studies in cancer prevention and interception, both individual agents and combination interventions. Given that findings from many preclinical studies evaluating individual agents have generally not been confirmed in human studies, our focus with individual NPs in this review is on studies involving humans, especially clinical trials. Fewer combination intervention studies have been conducted, so we have broadened our review to include preclinical studies. We conclude with how the Division of Cancer Prevention (DCP) within the NCI is providing funding to encourage the research community to propose natural product studies in cancer prevention and interception to advance the field.

## 1. Introduction

Natural products (NPs) have provided remarkable medications for disease prevention and treatment, such as cocaine and codeine for pain relief, digoxin to treat cardiac arrhythmias, quinine and artemisinin for malaria, salicylic acid for inflammatory conditions, and silymarin and pilocarpine to treat dry mouth, glaucoma, and liver diseases. Two agents for cancer treatment derived from NPs include vincristine from the periwinkle plant, which is used to treat certain leukemias and lymphomas, and taxanes from the bark of the Pacific yew tree and the hazel plant, which are used to treat lung, ovarian, and breast cancer. This report is a follow-up to our earlier publication [1], which focused on cancer prevention and treatment with combination NPs. In the follow-up, the objective is to address topics that were not addressed well, including single-agent NP preclinical studies, combination NP clinical studies, and the National Cancer Institute (NCI) Division of Cancer Prevention (DCP) funding opportunities, to identify new efficacious NPs. This report is not meant to be exhaustive but rather representative of studies relevant to the topic addressed in each section.

## 2. Single Agents

Most NP research, both preclinical and clinical, has evaluated single agents. Animal studies are easier, cheaper, and quicker to perform. They provide important insights regarding the efficacy of the agent in a living organism. While not meant to be exhaustive, we highlight several relevant NP studies in rodents in this section. Unfortunately, many findings from preclinical studies have not been replicated in humans. For this reason, we highlight human-focused studies of single NPs. For the purposes of this review, single agents may include a single compound or natural mixture of compounds (extract).

### 2.1. Ingenol Mebutate

Ingenol mebutate (IM), derived from the plant *Euphorbia peplus* L, was developed to treat actinic keratosis (AK). It works by a dual mechanism of action: (1) a rapid induction of cell death and (2) a delayed neutrophil-mediated cellular cytotoxicity response [2]. IM is an FDA-approved topical treatment for AK. IM has also been used off-label to treat multiple other skin disorders, including squamous cell carcinoma in situ [2]. On the other hand, the agent was withdrawn from the EU market in 2020 based on evidence that the agent may increase the risk of squamous skin cancer.

### 2.2. n-3 Fatty Acids (n-3FA)

A systematic review across 20 cohorts demonstrates heterogeneity regarding the results of n-3FA on cancer risk [3]. Of the eleven breast cancer studies evaluated, one demonstrated increased and three lowered risk and seven had no significant association. Seventeen of the eighteen colorectal cancer studies had no significant effect.

Conclusions: The literature spanning clinical trials and cohort studies is not consistent with a significant association between omega-3 fatty acids and cancer incidence. The currently available data are, at best, mixed regarding their potential benefit in the prevention of cancer [4].

### 2.3. Allium Compounds

These compounds are derived from garlic, onions, shallots, chives, and leeks [5]. A variety of possible anticarcinogenic mechanisms have been proposed for allium compounds to prevent cancer [6]. The strongest evidence for cancer prevention with allium compound use was in gastrointestinal cancers [5]. A meta-analysis of studies evaluating the impact of allium compounds on gastric cancer found that comparing the highest to lowest consumption groups, there was a 46% reduction in risk [7]. A network of case–control studies in Italy and Switzerland involving over 3000 individuals found that both onions and garlic were protective against cancers of the colon and rectum [8].

### 2.4. Vitamin D

Vitamin D is the precursor of calcitriol (1,25-dihydroxyvitamin D3:1,25(OH)2D), which regulates the expression of multiple genes [1]. Epidemiologic studies suggested differential effects on cancer risk based on organ site. Higher vitamin D levels in the blood have been associated with a reduced risk of colorectal cancer [9], and vitamin D deficiency is associated with increased bladder cancer risk [10]. On the other hand, higher vitamin D levels are associated with nonaggressive but not aggressive PCa [11].

### 2.5. Carotenoids

While epidemiologic and animal data provide evidence that carotenoids protect against cancer, human randomized controlled trials are conflicting. Arguably, the two most important human trials involved two agents. As such, the studies are, therefore, discussed below in the section on combination strategies.

#### Lycopene

Lycopene is a carotenoid. A potential role for lycopene in cancer prevention has focused on PCa risk, with many studies observing that both dietary intake and circulating levels of lycopene were directly associated with a reduced risk of PCa [12]. A six-month intervention of lycopene and green tea dietary advice or supplementation intervention in 128 men with elevated PSA levels was conducted on patients who were cancer-free. Increased lycopene intake altered the serum metabolome of men at risk for PCa. Lycopene lowered levels of pyruvate, which suggests that lycopene may be causally related to reduced PCa risk [13].

### 2.6. Perillyl Alcohol (PA)

PA is a monoterpene. Enthusiasm for this agent in cancer prevention has recently waned, in large measure because the encouraging findings from preclinical studies have not translated into clinical effect. For example, an NIH-funded trial of actinic keratoses, a precancerous skin lesion, evaluated the benefit of two different doses of PA cream vs. cream lacking PA and did not demonstrate a significant benefit (Clinicaltrials.gov: NCT0060834)

### 2.7. Melatonin

Preclinical studies support a potential chemopreventive effect of melatonin, an acetamide. Epidemiologic findings regarding dose–response using the agent were mixed, with some showing a lower risk, and others showed an increased risk [14]. More clinical trial data are available using melatonin as a chemopreventive agent in combination with other agents in patients with cancer.

### 2.8. Sulforaphane

Sulforaphane is an isothiocyanate. Preclinical studies of the agent found in broccoli and other vegetables in the *Brassicaceae* family suggest multiple mechanisms to prevent cancer. Animal studies suggest a modest chemopreventive effect with little to no toxicity [15]. A clinical trial enrolled 76 women who consumed either sulforaphane or control for 2–8 weeks prior to biopsy for an abnormal mammogram. SFN decreased cell proliferation (*p* = 0.003), as measured by Ki-67 [16].

### 2.9. Tea (Green, Black)

A Cochrane review evaluated 142 completed and two ongoing studies to assess the potential chemopreventive efficacy of green tea. Eleven studies, including 1795 participants receiving green tea extract or a placebo, were deemed to be high quality. PCa risk was decreased by 50% overall and had a wide confidence interval. The risk was increased by 50% for gynecologic cancer, again with a wide confidence interval. There was no effect on melanoma. Relatively common adverse effects included GI, including an increase in liver enzymes [17]. Among cohort and case–control studies, there was an overall 17% lower risk of cancer overall, again with a wide confidence interval. In short, conflicting results were conducted primarily in Asian populations, so generalizability is likely not possible.

Fewer clinical studies have evaluated the chemopreventive benefit of black compared to green tea. Of three cohort studies, two found no association between black tea consumption and colorectal, breast, lung, stomach, or renal cell cancer risk, while one study found an ovarian cancer protective effect with the consumption of two or more cups of black tea [18]. In addition, clinical studies found a lower risk of endometrial and skin cancer among those who drank two or more cups of black tea vs. those who did not.

#### Polyphenol E (Poly E)

Poly E, derived from green tea, has been evaluated as a cancer-preventive agent. Poly E is a mixture of green tea components: 65% of epigallocatechin gallate (EGCG) along with epicatechin. While preclinical studies have been encouraging, this has not translated into human efficacy. Genital warts are a concerning lesion in the anogenital area. They are caused by human papillomavirus. Poly E was shown to clear genital warts in 59% vs. 37% of control participants for patients administered a placebo. Poly E is now FDA-approved to treat genital warts [19]. Patients with ulcerative colitis (UC), an inflammatory disease of the colon, have a six times greater risk of developing colorectal cancer than those of average risk. Twenty patients with ulcerative colitis were randomized to oral Poly (400 mg or 800 mg daily) or placebo in a double-blinded placebo-controlled pilot study. After 56 days of therapy, 66.7% (10 of 15) in the Poly E group vs. 0% in the placebo group responded. The remission rate was 53.3% vs. 0% for the placebo with only minor side effects. On the other hand, it was not found to be effective in decreasing aberrant crypt foci [20].

### 2.10. Isoflavones

A prospective study enrolled thirty-four healthy premenopausal women randomized to 40 mg or 140 mg isoflavones daily through one menstrual cycle. Intraductal and blood specimens were collected to evaluate breast-specific and systemic effects of each intervention. Cytology did not significantly change at either isoflavone dose. Serum levels of the estrogenic marker C3 posttreatment were inversely related to changes in serum genistein (*p* = 0.0045) in women consuming low but not high doses of isoflavones. The *RARβ2* hypermethylation increased posttreatment and was correlated with the posttreatment genistein level considering the entire group (*p* = 0.0017) and those receiving a high dose of isoflavones (*p* = 0.021). Isoflavones induced gene methylation changes, which correlated with genistein levels. The inverse correlation between C3 and genistein suggests an antiestrogenic effect, while isoflavones induced dose-specific changes in *RAR β2* and *CCND2* gene methylation, two cancer-related genes [21].

### 2.11. Curcumin

A phase IIA clinical trial enrolled subjects to receive either 2 or 4 g of curcumin daily to determine whether curcumin could prevent the formation of aberrant crypt foci (ACF), reduce eicosanoids, and inhibit cell proliferation. Those taking 4 g of curcumin daily had a 40% reduction in ACF, whereas a separate clinical trial evaluating individuals with familial adenomatous polyposis (FAP) receiving 3 g of curcumin daily or a placebo did not find an effect on the number of colorectal polyps or polyp size [22]. A small 6-month study incorporating curcumin and quercetin found a 60% reduction in polyp number and a 51% decrease in polyp size [22].

### 2.12. Selenium

Small studies found encouraging results with selenium to prevent cancer. These results led to the Selenium and Vitamin E Cancer Prevention Trial (SELECT), in which selenium supplementation did not lower overall PCa risk but increased the risk of high-grade disease and type 2 diabetes [23]. A Cochrane systematic review of randomized controlled trials involving 27,232 participants randomized to selenium supplementation vs. control in preventing cancer found no beneficial effect [24]. Another study evaluated single-nucleotide polymorphisms (SNPs) associated with toenail and circulating (TAB) selenium levels with 22 site-specific cancers or any cancer [25].

## 3. Combination Strategies

Compound combinations, with one or more of the agents coming from a natural source, may increase or decrease chemopreventive efficacy. Combination strategies involve the investigation of various natural substances, such as herbs, botanicals, dietary supplements, and other non-pharmaceutical NPs, to reduce the risk of lesion initiation or progression of the lesions to invasive cancer. It is important to recognize that there are both plant, marine, and animal-derived NPs. Combination studies explore whether combining NPs that interact with different targets can provide enhanced protective effects through synergistic or additive chemopreventive effects. Combination chemopreventive chemical agents produce a stronger protective effect in colon cancer both preclinically and in clinical studies [26,27,28]. Examples include the combinations of difluromethylornithine plus sulindac and sulindac plus erlotinib. There is increasing evidence that a single NP may not be optimally effective in cancer prevention or the interception of high doses that may be toxic, whereas combination interventions using lower doses with no or lower toxicity might work. Apart from providing efficacy, combination NP strategies might boost the immune system and overcome potential side effects. In this section, we describe combination strategies using NPs for cancer chemoprevention. Human studies using combination interventions, especially randomized prospective studies, are limited. For this reason, we discuss both preclinical and human studies.

### 3.1. Preclinical Animal Model Studies for Combination Chemoprevention

Although a number of chemoprevention efficacy studies using individual NPs (dietary, marine, plant-based, etc.) have been evaluated, not many combinational studies have been conducted both in preclinical in vivo and clinical studies. Importantly, animal studies help determine if the combinations are synergistic, additive, or antagonistic and if there is increasing synergy with increasing or decreasing agent doses. The reported in vivo studies using animal models, such as mice or rats, to mimic human conditions and test the efficacy of various NPs in preventing the development of cancer or inhibiting its progression along with changes in the biomarker expression are summarized (Table 1).

#### 3.1.1. Colon Cancer

Quercetin and resveratrol have been shown to be effective natural agent combinations in a few studies. In an azoxymethane (AOM)-induced rat colon cancer model, a resveratrol (8 mg/kg) and quercetin (10 mg/kg) combination showed a better inhibitory effect on histopathological changes, apoptosis induction, and cell proliferation than individual agents. Importantly, a high-grade crypt abnormality was observed in 73% of control animals, 45% of those treated with resveratrol, 36% in the quercetin group, and 27% in the combination-treated animals [29]. Hu et al. evaluated the combination chemopreventive effects of dietary selenium (1 ppm) and green tea extract (0.5%) in the AOM-induced rat colon cancer model [30]. Dietary combination treatment significantly inhibited large ACF, tumor incidence, multiplicity, and size (*p* < 0.01), with a reduction in cell proliferation, cyclin D1, and DNMT, the restoration of SFRP5 mRNA, and the induction of histone H3 acetylation. The combinations were more effective in showing additive effects than individual agents [30]. Further, epigallocatechin gallate (EGCG), combined with the colon cancer chemopreventive agent sulindac, demonstrated significant inhibition (76%) of intestinal polyps in mice with a mutant (APC) locus [31]. Bose et al. showed that the combination of EGCG and fish oil reduced intestinal polyp numbers by 53% in the APC min mice model with an increase in apoptosis and reduced PGE_2_ levels [32]. In the 1,2 dimethylhydrazine rat colon cancer model, curcumin and catechins significantly lowered the incidence of colon tumors compared to individual and control group rats. Tumor inhibition was associated with a lower proliferative index and increased apoptosis and had a greater effect in the combination than in individual agent-treated groups [33]. In another study, an AOM-induced rat colon cancer model was used to determine the chemopreventive effects of garlic and tomato suspensions (aqueous) individually or in combination. Results showed a significant inhibition of aberrant crypt foci in all treatment groups, with additive effects in the combination treatment group (71.6%). A significant reduction in cell proliferation, apoptosis induction, and the suppression of COX-2 expression was observed in the combination treatment groups compared to individual treatment arms [34]. Velmurugan et al. evaluated the combination chemopreventive effects of S-allylcysteine (SAC) and lycopene in a carcinogen-induced rat gastric cancer model. Each agent individually suppressed gastric cancer development, and the combination was more effective [35]. The AOM-induced colon cancer studies in SD rats or mice with the combination of fish oil and pectin reduced ACF, tumor incidence, and multiplicity in several studies [36,37,38,39]. Sulforaphane and indole-3-carbinol have been investigated for their combined chemopreventive properties against various cancers in vitro. In in vivo studies, dietary administrations of a combination of sulforaphane (300 ppm) and dibenzoyl methane (0.5%) significantly inhibited the development of intestinal polyps 57% (*p* < 0.001) and blocked the colon tumor development in the APC min mouse model. The treatments also resulted in decreased levels of PGE_2_ and LTB4, lower cell survival, and the inhibition of growth-related signaling pathway and biomarkers in intestinal polyp biomarkers [40].

#### 3.1.2. Head and Neck Cancer

Combining curcumin from turmeric and green tea extract has been studied for their potential chemopreventive effects. In a study, curcumin combined with metformin in 4NQO induced the mouse model of oral cancer and significantly reduced tumor volume and improved overall survival (*p* = 0.03) by downregulating cancer stem cell markers in the treated groups [41]. Using the hamster buccal pouch carcinoma model, Saleh et al. demonstrated that the curcumin and green tea (EGCG) combination was superior to individual agents when treated for 18 weeks in inhibiting oral tumorigenesis and inducing apoptosis [42]. Similarly, this combination showed decreased proliferation and increased apoptotic indices in the dysplasia and oral SCC, suggesting that the combination efficacy is seen in the post-initiation stages of carcinogenesis, supporting the idea that this could be an effective cancer interception strategy [43]. In a xenograft head and neck cancer model, Amin et al. found that the combination of resveratrol and EGCG demonstrated synergistic a chemopreventive effect due to inhibition of the AKT-mTOR pathway and increased apoptosis [44].

**Table 1:** Studies on the in vivo efficacy of natural product combinations.

Cancer Type	Agent Combination	Animal Model	Efficacy	Potential Mechanisms/Targets	Reference
Colon	Quercetin (8 mg/kg) + Resveratrol (10 mg/kg)	AOM-induced rat colon cancer	High-grade crypt abnormality in control: 73%, resveratrol: 45%, quercetin: 36%, combination tx: 27%	↑ apoptosis, ↓ cell proliferation	[29]
Colon	Selenium (1 ppm) + Green Tea Extract (0.5%)	AOM-induced rat colon cancer	Combination of tx-inhibited large ACF, tumor incidence, multiplicity, and size (*p* < 0.01)	↓ cell proliferation, cyclin D1, DNMT, restoration of SFRP5 mRNA, ↑ histone H3 acetylation	[30]
Intestine: multiple sites	EGCG (0.1%) + Sulindac (0.03%)	APC min mice	Tumor#/mouse in untreated control, EGCG, and Sulindac groups were 76, 57, and 49, respectively The combination tx group had only 32 tumors (~66% reduction, *p* < 0.05)	ND	[31]
Intestine: multiple sites	Fish Oil (12%) + EGCG (0.16%)	APC min mice	Combination tx reduced total tumor multiplicity by 53%, *p* < 0.05	↑ apoptosis ↓ PGE2 levels	[32]
Colon	Curcumin (0.1%) + Catechin (0.1%)	DMH-induced rat colon cancer	ACF number and colon tumor incidence decreased, respectively, by 57% and 53% in the combination tx group compared to untreated control	↓ proliferative index ↑ apoptosis	[33]
Colon	Garlic (2%) + Tomato (2%)	AOM-induced rat colon cancer	Tx resulted in a significant reduction in ACF by 45% in garlic, 68% in tomato, and 72% in the combination tx groups	↓ cell proliferation ↑ apoptosis ↓ COX-2 expression	[34]
Gastric	S-allylcysteine (100 mg/kg) + Lycopene (1.25 mg/kg)	MNNG and S-NaCl-induced gastric carcinogenesis in rats	Combination tx reduced tumor incidence from 100 to 17% with the tumor burden lowered from 148 to 24 mm	↓ Bcl-2, ↑ Bax, ↑ Bim ↑ caspase 8	[35]
Colon	Fish Oil (11.5%) + Pectin (6%)	AOM-induced rat colon cancer	Combination tx had a significantly lower colon tumor incidence (51%) compared with those receiving the control diet (76%) (*p* = 0.016)	↑ Bcl-2 promoter methylation ↑ apoptosis	[36]
Colon	Fish Oil (11.5%) + Pectin (6%)	AOM-induced rat colon cancer	Combination tx protected the colon from the carcinogen-induced dysregulation of multiple miRNAs	differential expression of miRNAs (Let-7d, miR-15b, miR-107, miR-191, miR-324-5p)	[37]
Colon	Fish Oil (11.5%) + Pectin (6%)	AOM-induced colon cancer in Lgr5-EGFP-IRES-creERT2 mice	Total ACF in the control vs. tx group: 44 vs. 28 (*p* < 0.05), multi-crypt ACF 6 vs. 4 (*p* = 0.06)	↑ miR-19b, miR-26b, miR-203 in Lgr5high cells	[38]
Colon	Fish Oil (11.5%) + Pectin (6%)	AOM-induced rat colon cancer	Combination tx vs. control significantly reduced high multiplicity aberrant crypt foci from 63.2 to 26.7	upregulation of lipid catabolism and beta-oxidation-associated genes	[39]
Intestinal tumorigenesis	Sulforaphane (300 ppm) + Dibenzoylmethane (0.5%)	APC min mice	Combination tx inhibited intestinal polyp formation by 57% (*p* < 0.001) and completely prevented tumor development (*p* = 0.002)	↓ PGE2, ↓ LTB4	[40]
Oral squamous cell carcinoma	Green Tea (6 mg/mL) ingested orally + Curcumin (10 mmol) applied topically	DMBA-induced buccal pouch carcinoma in hamsters	Green tea and curcumin combination inhibited oral tumorigenesis and induced apoptosis	↓ cancer stem cell markers (CD133, CD44)	[42]
Oral squamous cell carcinoma	Green Tea (6 mg/mL) ingested orally + Curcumin (10 mmol) applied topically	DMBA-induced oral carcinogenesis in hamsters	Combination tx decreased precancer and SCC lesion numbers by over 50% and lesion volume by one-third for precancers and two-thirds for cancers	↑ apoptosis ↓ proliferation	[43]
Head and neck	Resveratrol (30 mg/kg) + EGCG (125 mg/kg)	Tu212 xenograft model	Tumor weight and volume were significantly reduced by combination tx	↓ AKT-mTOR pathway ↑ apoptosis	[44]
Prostate	Vitamin E (800 IU) + Selenium (200 µg) + Lycopene (50 mg)	Lady (12T-10) transgenic mouse model	Combination tx reduced the incidence of PCa by >80%	↑ apoptosis ↓ proliferation	[45]
Prostate	Curcumin (6 μmol i.p.) + PEITC (5 μmol i.p.)	PC-3 PCa xenograft model	Combination tx significantly reduced tumor volume vs. individual tx and control groups	↓ proliferation ↑ apoptosis	[46]
Prostate	Tomato (5%) + Broccoli (5%)	Dunning R3327-H PCa rat model	Combination tx decreased the tumor weight by 52% (*p* < 0.001)	↓ proliferation ↑ apoptosis	[47]
Lung	I3C (10 μmol/g diet) + Silibinin (7 μmol/g diet)	NNK-induced lung cancer in A/J mice	Lung adenocarcinoma presence and tumor number were reduced by 60% and 95%, respectively	↓ p-Akt, ↓ p-ERK ↓ cyclin D1 ↑ apoptosis	[48]
Breast	SFN-enriched Broccoli Sprouts (13% in diet) + Genistein (250 mg/kg diet)	C3(1) SV40 Tag transgenic mouse model	Combination tx was more effective at reducing tumor incidence and volume compared to the control and either single treatment	ND	[49]
Breast	Genistein (250 mg/kg) + Tamoxifen (25 mg/pellet) implanted subcutaneously	C3(1)-SV40 Tag transgenic mouse model	The tumor growth rate was reduced by combination tx	↓ tumor cell proliferation	[50]
Pancreas	Curcumin (2000 ppm) + Fish Oil (15%)	BxPC-3 pancreatic cancer xenograft model	Combination tx reduced tumor volume > 72%	↓ COX-2, ↓ iNOS ↓ 5-LOX ↑ p21	[51]

Abbreviations: AOM: azoxymethane; APC: adenomatous polyposis coli; DMBA: 7,12-dimethylbenz[a]anthracene; ECGC: epigallocatechin gallate; NNK: nicotine-derived nitrosamine ketone; PCa: prostate cancer; PEITC: phenethyl isothiocyanate; SCC: squamous cell carcinoma; Tx: treatment; ND: not determined; ↑: increase; ↓: decrease.

#### 3.1.3. PCa

The combination of vitamin E, selenium, and lycopene inhibited PCa development, reduced proliferation, and induced apoptosis in a transgenic mouse model when administered at the initiation stages [45]. In a PCa xenograft model, Khor and colleagues showed the combined inhibitory effects of curcumin and phenethyl isothiocyanate with the suppression of proliferation and tumor growth and the induction of apoptosis [46]. Using a Dunning R3327-H-prostate tumor model, the combination of freeze-dried tomato and broccoli vs. food alone significantly enhanced anti-tumor activity, as evidenced by decreased tumor weight (52%; *p* < 0.001), reduced proliferation, and increased apoptosis [47].

#### 3.1.4. Lung Cancer

The combination of indole-3-carbinol (I3C) and silibinin reduced lung tumor multiplicity by 60% compared to weaker reductions in individual groups (I3C 43%; silibinin 36%) in the carcinogen-induced lung cancer mouse model. Further, the adenoma and adenocarcinoma numbers per mouse were reduced by 92% and 95% with the combination treatments. The protein expression of genes associated with proliferation (*p-Akt*, *p-ERK*, *cyclin D1*) was reduced, and apoptosis increased vs. control and individual treatments [48].

#### 3.1.5. Breast Cancer

In the C3(1) SV40 TAg transgenic mouse model, the combination of SFN-enriched broccoli sprouts (13% in the diet) and genistein (250 mg/kg diet) was more effective in preventing breast tumors (extending tumor latency, reducing tumor volumes and sizes) compared to single agents [49]. In the same animal model, dietary genistein enhanced the efficacy of tamoxifen in reducing ER breast tumors [50].

#### 3.1.6. Pancreatic Cancer

In a pancreatic cancer xenograft model, the combination of fish oil (omega-3 fatty acids) and curcumin was evaluated. The combination treatment resulted in a >72% (*p* < 0.0001) reduction in the tumor volume with a decrease in the expression of COX-2, iNOS, and 5-LOX and an increased p21 expression in the treated xenograft tumors [51].

**Figure 1:**
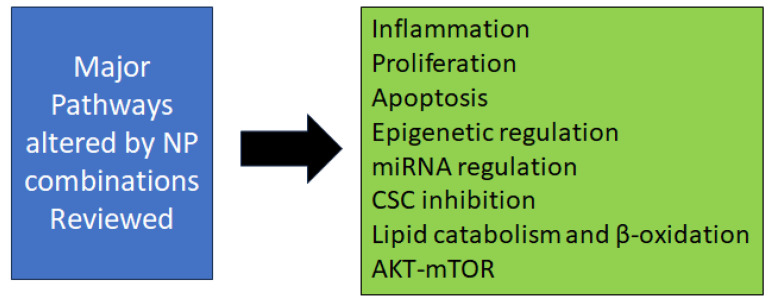
The major pathways and mechanisms altered by natural product combinations examined in this review.

Although the above preclinical in vivo studies across organ site cancers showed significant combinatory chemopreventive efficacies along with changes in biomarker expression and pathway modulations (Figure 1), further studies are warranted to evaluate their long-term agent toxicity followed by IND-enabled studies before moving them to clinical trials.

### 3.2. Clinical Studies for Natural Product Combination Chemoprevention

It is important to note that while animal studies provide valuable insights into the potential of naturally occurring agents for cancer prevention and interception, results in animals do not always directly translate to human outcomes. Human studies are necessary to confirm the effectiveness and safety of these combinations for cancer prevention and interception in humans. Additionally, the choice of natural agents and their combinations may vary depending on the specific type of cancer being studied. However, there is a paucity of human studies, especially randomized prospective clinical trials. Ensuring the safety and quality of NPs used in these trials is essential. Participants should be monitored for adverse events, and regulatory guidelines and quality control standards must be followed. There are mixed results in the clinical trials, which somewhat reduced the enthusiasm in the field. Further, there are very few reports on the combination chemoprevention studies in humans, as summarized in this section. Representative studies are outlined in Table 2.

The SELECT clinical trial tested the individual and combination chemopreventive effects of oral selenium and vitamin E supplementation in healthy volunteers against PCa development. SELECT was stopped early both because of safety concerns and negative data [52]. Another negative clinical trial was the ATBC study, which evaluated the individual and combinatorial effects of α-tocopherol (vitamin E) and β-carotene against lung and other cancers [53]. This randomized study enrolled over 29,000 male cigarette smokers who received test agents vs. placebo for five years. The study demonstrated that β-carotene participants had an increase in lung, prostate, and stomach cancer. In the vitamin E group, there was a lower incidence of PCa and CRC and an increase in stomach cancer. Participants who received both beta-carotene and vitamin E supplements had a slightly higher risk of lung cancer compared to those who did not receive the supplements. This unexpected result raised concerns about the safety of these supplements, particularly for smokers. It is important to note that the ATBC trial has had a lasting impact on the field of nutrition and cancer prevention research. The study results highlighted the complexity of the interactions between vitamins, health outcomes, and especially supplements, which are ingested generally at higher doses than are consumed in the diet. The CARET (Beta-Carotene and Retinol Efficacy Trial) investigated the effects of beta-carotene (30 mg per day) and retinol (vitamin A; 25,000 IU) on health outcomes, primarily in individuals at a high risk of lung cancer. Similar to the ATBC trial, the CARET found that the combination of beta-carotene and retinol supplements did not reduce lung cancer risk, with a trend toward an increased risk of lung cancer among those who received the supplements, particularly among current smokers. Perhaps most concerning was an increased overall mortality rate in the group receiving the combination of beta-carotene and retinol. Both ATBC and CARET highlighted potential interactions between smoking and the use of beta-carotene supplements [54]. Both studies provided beta-carotene at doses 10–20 fold higher than is generally consumed. Therefore, currently available data do not support a risk reduction with carotenoid use in humans.

In the Systematic Evaluation of Aspirin and Fish Oil polyp prevention trial, eicosapentaenoic acid (EPA) plus aspirin did not show a chemopreventive effect at the 1-year surveillance colonoscopy [55]. In a double-blind randomized study, curcumin, green tea extract, or both were administered to individuals with oral potentially malignant disorders, demonstrating a downregulation of molecular biomarkers (Ki67, cyclin D1, and p53) at 12 weeks. The clinical response rate was higher in the combination group (65%) vs. individual groups (55% for curcumin and 35% for green tea extract groups) with a statistically significant downregulation (*p* < 0.01) of molecular biomarkers. [56]. Based on preclinical efficacy and its preferential distribution in colonic tissue, curcumin + quercetin was evaluated in APC patients. After six months of treatment vs. baseline, participants treated with the combination had lower polyp number and size (*p* < 0.05) and minimal side effects [57]. A phase II trial of men with PCa and rising prostate-specific antigen (PSA) levels suggested that a combination of lycopene and soy isoflavones stabilized PSA levels [58]. Combination treatment with either garlic (aged + garlic oil) or vitamin/mineral supplements (vitamins C and E and selenium) for 7.3 years showed a significant reduction in death from gastric cancer, and the incidence decreased with vitamin/mineral but not the garlic combination [59]. Additional NP combination clinical trials (modified from Sauter [1]) are outlined in Table 2.

## 4. Challenges When Conducting NP Studies

High-risk cohorts, such as individuals with hereditary syndromes, precancer lesions, and those exposed to environmental or occupational carcinogens, are typically at an increased risk of cancer. The hope is that cancer prevention and interception approaches for these individuals using NPs will provide beneficial chemopreventive effects without undue adverse effects. While there is great potential in the discovery and development of NPs for cancer prevention and interception, several gaps and challenges exist in this field. One of the biggest challenges with NP studies, since NPs can have variable components, depending on how and where the product was produced/grown, is to optimize the reproducibility of the studies and agents. NPs come from diverse sources, making it difficult to standardize their composition, purity, and dosage. Further, conducting combination NP cancer prevention studies can be challenging due to potential interactions between different NPs, variability in product quality, and the need for a large sample size to detect meaningful differences in cancer prevention and interception efficacy outcomes. NP studies can yield inconsistent results, partly due to variations in study design, patient populations, and NP dose. This makes it challenging to draw clear conclusions about NP efficacy. The lack of cancer prevention and interception randomized controlled trials evaluating combination NPs with cancer incidence or biomarker changes is a major shortcoming, and more studies are needed. Biomarker changes can provide insights into the mechanisms of action and potential side effects of NPs. Following in-depth preclinical animal model studies using multiple agents targeting multiple pathways in human clinical trials, it is imperative that NP chemopreventive agents be tolerated, lack adverse drug–drug interactions, and be readily bioavailable and safe after long-term use [60]. A treatment combination with multiple low-dose NP agents, or a low dose of NPs combined with a chemical agent, may allow this. While the effectiveness of combinations can vary and many factors can influence the outcomes, in vivo models will aid in assessing the efficacy of alternative dosing strategies and routes of treatment combinations to reduce toxicities while maintaining efficacy. Other important factors to consider for NP combination studies include agents’ bioavailability and a comprehensive understanding of their mechanisms of action, the microbiome, biotransformation, dose optimization, agent interactions, statistical and alternative intelligence models, potential complementary mechanisms, and the potential to overcome antagonistic activities and adverse effects.

Novel approaches to enhance the bioavailability of NPs are necessary to enhance their chemoprevention potential. One way to achieve this is by combining agents that increase the bioavailability of other agents. For example, curcumin was observed to increase the permeability and bioavailability of EGCG, suggesting that the P-glycoprotein pump inside the intestine can enhance EGCG permeability [61]. Combination NP consumption supports public health recommendations by increasing the intake of a variety of plant components. Combination NP studies can pave the way for future cancer prevention and interception clinical trials with new perspectives.

Several efforts are underway to enhance the bioavailability of NPs. Resveratrol, although an effective chemopreventive agent, was found to be rapidly metabolized and possessed poor bioavailability, hindering its translatability to humans. Similarly, emodin, a traditional medicine, showed low bioavailability in preclinical studies. One of the reasons for its poor bioavailability is its rapid glucuronidation in the liver and intestine. In order to slow down or inhibit the glucuronidation process, combination studies were conducted using piperine as a bioenhancer. When resveratrol (100 mg/kg) was combined with piperine (10 mg/kg), the bioavailability of resveratrol was significantly improved [62]. Similarly, piperine at 20 mg/kg significantly enhanced the bioavailability of emodin by inhibiting the glucuronidation process [63]. Further studies will shed light on the exact mechanisms through which piperine enhances the bioavailability of other NPs.

Challenges when conducting NP studies can lead to negative results. NPs may demonstrate cross-resistance and overlapping side effects. Combinations should target multiple pathways or the same pathway through multiple mechanisms to maximize efficacy while limiting toxicity. Unfortunately, achieving this combination of optimization has not always been successful. As a tumor progresses, the tumor cells within it become more heterogeneous. NP interactions may work together synergistically in an additive fashion or an antagonistic fashion. One NP may potentiate a second, which may increase efficacy, toxicity, or both.

Finally, understanding the long-term effects of using NP agents for cancer prevention and interception is often lacking. Monitoring individuals over extended periods is necessary to assess their safety and efficacy.

## 5. Potential Opportunities for the Discovery and Development of NPs for Cancer Prevention and Interception

To address gaps and challenges in NP prevention and interception research, the NCI DCP has created funding opportunities to encourage collaborative efforts within the research community. These Notices of Funding Opportunity (NOFOs) encourage projects to close gaps and overcome challenges in this important area of research.

Most NPs are non-specific and show pleiotropic effects, in that they bind to numerous targets. There is an urgent need for (1) better NP libraries to produce better results and (2) screening to identify new modalities to change the current trajectory of cancer prevention and interception research. Unique resources available from the NCI may overcome deficiencies of historical approaches by providing quality-controlled samples that are associated with substantial informatics support to improve the ability to select NPs that can provide clinical benefit. The NCI has one of the world’s largest, most diverse collections of NP extracts (>500,000 fractions) collected from various plant, marine, and microbial sources. These NP libraries are readily available for use by the research community at no cost.

The NCI DCP has addressed gaps in knowledge through NOFOs to identify new efficacious NPs (Table 3). A recently launched NOFO, the “Discovery and Development of NP for Cancer Interception and Prevention Program DDNP-CIP”, supports the discovery and development of new NPs that are safe, non-toxic, and efficacious for cancer interception and prevention. DDNP-CIP is supported through the newly published notice of funding opportunity, https://grants.nih.gov/grants/guide/rfa-files/RFA-CA-23-028.html (accessed on 17 January 2024) which intends to fund UG3/UH3 exploratory/developmental projects. The specific purpose of the first (UG3) phase is to identify clinically relevant targets and develop and validate assays for bioactivity and toxicity screening of the natural compounds. The development of high-throughput screening (HTS) amenable assays that can predict a desirable cancer interception endpoint is a continued area of need. Proposed studies under the UG3 phase should focus on the development of primary and secondary assays and HTS strategies that meet robust HTS requirements. The specific purpose of the second (UH3) phase is to screen NP libraries, with full-scale characterization, efficacy testing, and the development of the screened agents. Grant applicants’ projects with clinically relevant cancer interception pathways and targets can take advantage of NCI’s large library of “ready-to-screen” pre-fractionated NPs to speed up bioassay-directed isolation and characterization of the most promising ones. Applicants can also propose to use commercial libraries, investigator-developed libraries, and robust HTS strategies. New natural agents discovered will move to the existing advanced preclinical development program, PREVENT, for further development toward early-phase cancer prevention clinical trials by DCP CP-CTNet.

The NCI PREVENT Cancer Preclinical Drug Development Program (PREVENT) supports the preclinical development of innovative cancer prevention and interception interventions and biomarkers for clinical trials. PREVENT’s current research priority areas include immunoprevention, chemoprevention, and clinically translatable biomarkers (https://prevention.cancer.gov/major-programs/prevent-cancer-preclinical-drug-development-program-prevent (accessed on 17 January 2024)). PREVENT projects investigate agents for cancer chemoprevention, some of which are NPs. Some of the NP-relevant projects supported by the PREVENT program include the Preclinical Development of Newly Formulated Chemopreventive Agent 4-methylumbelliferone Prodrug (261201500036I-0-26100010-1), the Use of Rosemary Extract/Carnosic Acid for Prevention of Ductal Carcinoma in situ (75N91019D00016-0-759102000001-1), TP-252: A Longer Acting Eicosapentaenoic Acid (EPA) Analogue for Colorectal Cancer Chemoprevention (75N91019D00019-0-759101900132-1), Chemoprevention with mitochondria-targeted honokiol in mouse models of lung cancer: adenocarcinoma and squamous cell carcinoma, and the Preclinical Evaluation of a New Lipid-Based SMEDDS BR-9001 Formulation (261201500042I-0-26100003-1, 261201500042I-0-26100003-1). Further, the NCI DCP (https://prevention.cancer.gov/about-dcp (accessed on 17 January 2024)) supports the testing of NPs in phase I/II clinical trials. For example, DCP is currently investigating “Testing the Effect of the Broccoli Seed and Sprout Extract, Avmacol ES, on the Cancer Causing Substances of Tobacco in Heavy Smokers”. This phase II trial tests whether broccoli seed and sprout extract work to break down cancer-causing substances in tobacco in heavy smokers. Smokers are at an increased risk for developing lung, head, neck, and other cancers. Broccoli seed and sprout extracts may break down and remove toxic substances caused by tobacco use and produce substances that protect cells from tobacco smoke-induced damage in current smokers.

Other NCI DCP NP NOFOs for cancer prevention and interception include the Notice of Special Interest (NOSI): Dietary Effects on Nutrient Sensing Pathways in Tumor Etiology and Prevention NOT-CA-21-121, the NOSI: Administrative Supplements for Validation Studies of Analytical Methods for Dietary Supplement Constituents NOT-OD-22-202, and the NCI Clinical and Translational Exploratory/Developmental Studies PAR-22-216.

## 6. Conclusions

NPs are an important source of compounds for cancer prevention and interception. Since a single NP may not be optimally effective in preventing cancer, NP combinations are being investigated with increased frequency in the hope of increasing chemopreventive efficacy. This study has limitations. It is not to be taken as an exhaustive review, nor does it cite every publication related to the NP discussed. On the other hand, it does address important NPs that have shown promise in cancer prevention and interception, and whenever possible, presents data demonstrating the prevention or treatment of one or more tumors, with less focus on biomarker studies. While most NP studies have not led to compounds that are useful for preventing cancer, there is a wide belief that there is value in the further investigation of NPs to identify effective compounds. The NCI has multiple funding programs to support the preclinical development of agents (PREVENT) and NP testing in phase I/II clinical trials. In addition, there are two NOSIs to encourage studies of NPs, one that addresses the effects of diet/cell interactions on early tumor development and a second that encourages submissions to validate analytic methods for dietary constituents.

## Figures and Tables

**Table 2:** Representative clinical trials evaluating combination natural products *.

Disease Endpoint	Gender	Interventions	Intervention Frequency/Length	Results (Efficacy, Targets, Mechanisms)
PCa prevention	♂ (≥50-AA; ≥55-others)	vitE 400 IU Selenium 200 mcg Placebo	Daily/7–12 years	↑ risk of PCa cancer: 1.6/1000 person-years for vitE, 0.8 for selenium, 0.4 with the combination vs. control
Lung and other cancer prevention in smokers	♂ 50–69	vitE β carotene Placebo	Daily/5–8 years	vitE had no effect on lung cancer incidence vs. control, while a lower incidence of PCa and colorectum was observed. Those receiving β carotene had an ↑ incidence of lung, prostate, and stomach cancer
High risk for esophageal and gastric cancer	♂ + ♀ 40–69	Retinol, zinc, riboflavin niacin, ascorbate molybdenum, vitE, β carotene, selenium, placebo	Daily/63 months	vitE (50 mg) + β carotene (15 mg) + selenium (50 mcg) ↓ mortality due to gastric cancer by 21% and total cancer mortality by 13%. Other nutrients: no significant effect
High risk for colorectal cancer	♂ + ♀ (55–73)	2 g EPA-free FA, 300 mg aspirin, both, or placebo	Daily/12 months	Neither EPA nor aspirin reduced colorectal adenomas
Oral potentially malignant disorders	♂ + ♀	Green tea extract (topical + 800 mg/d systemic, curcumin topical + 950 mg/d systemic, or both	Daily/3 months	Response (lower p53, Ki67, cyclin D1) ↑ in the combination group (65%) vs. curcumin (55%) or green tea extract (35%) (*p* < 0.01)
APC	♂ + ♀	480 mg curcumin 20 mg quercetin	Thrice daily/6 months	Combination tx led to ↓ polyp number and size (*p* < 0.05) after tx vs. baseline
PCa	♂	15 mg lycopene, 40 mg soy isoflavone, or both	Twice daily/6 months	Lycopene and combination tx led to stable PSA in 95% and 67%, respectively, in patients with previously rising PSA
PCa	♂ ≥50	Lycopene 30 mg Fish oil 1 g Placebo	Daily/3 months	No genes were significantly associated with a high intake of fish oil or lycopene at baseline or after 3 months of study
Gastric cancer prevention in an area where gastric cancer is endemic	♂ + ♀ 35–64	H pylori tx, garlic, vitamin C, E, selenium	Twice daily/7.3 years	Each tx: H pylori, garlic, vitamins C, E, selenium significantly ↓ gastric cancer mortality, incidence decreased with vitamin but not garlic supplements
Cancer and cardiovascular (CV) incidence and mortality	♂ 45–60 + ♂ + ♀ 35–60	vitC 120 mcg vitE 30 mg β carotene 6 mg selenium 100 mcg Zinc 20 mg Placebo	Daily/7.5 years	A 31% ↓ total cancer incidence and 37% reduction in all-cause mortality in men but not women vs. control
Prostatic intraepithelial neoplasia and suspicious prostate findings	♂ ≥21	Green tea extract Fish oil Placebo	Twice daily/up to 20 weeks	No significant ∆ in FA synthase or cell proliferation with green tea extract, fish oil, or the combination vs. control
Colorectal adenoma recurrence	♂ ≥50-AA; ≥55-others	vitE 400 IU Selenium 200 mcg Placebo	Daily/7–12 years	Neither selenium nor vitE affected adenoma recurrence vs. control
Smokers, former smokers, and workers exposed to asbestos	♂ + ♀ 45–69	β carotene 30 mg vitA 25,000 IU Placebo	Daily/4 years	β carotene and vitA may ↑ the risk of death from lung cancer, CV disease, and other causes
Postmenopausal women	♀ Post	CaCO_3_ 1000 mg vitD 400 IU Placebo	Daily/7 years	Ca and vitD: no effect on colorectal cancer incidence
Prevention of cancer and CV disease	♂ ≥50; ♀ ≥55	2000 IU vitD n-3 FA	Daily/5.3 years	Neither vitD nor marine n-3 FA significantly ↓ cancer or CV risk vs. control
Lung cancer prevention in former smokers	♂ + ♀ 40–80	Green tea beverage Polyphenon E Placebo	Daily/6 months	There was no significant effect on urinary 8-OHdG or 8-F2 isoprostanes with either treatment or control

* Modified from Sauter [1]. Some results were gleaned from publications, others were from Clinicaltrials.gov. All races enrolled in the listed studies. Abbreviations: APC: adenomatous polyposis coli; EPA: ecisopentanoic acid; PCa: prostate cancer; PSA: prostate-specific antigen; vit: vitamin; FAs: fatty acids. ↑: increase; ↓: decrease. Symbols: ♂: male; ♀: female.

**Table 3:** Opportunities for the discovery and development of NPs * for cancer prevention and interception.

NCI Program or Title of the Funding Opportunity	Notice of Funding Opportunity (Hyperlinks)	Funding Type	Submission Dates
DDNP-CIP program	RFA-CA-23-028	UG3/UH3	June 2023–2025
PREVENT program	PREVENT Concept Application	Contract	Twice a year, the second Monday in January and July
Dietary Effects on Nutrient Sensing Pathways in Tumor Etiology and Prevention	NOT-CA-21-121	NOSI	Various, NOSI expires September 2024
Administrative Supplements for Validation Studies of Analytical Methods for Dietary Supplement Constituents	NOT-OD-22-202	NOSI	Various, NOSI expires April 2025
NCI Clinical and Translational Exploratory/Developmental Studies	PAR-22-216	R21 Clinical Trial Optional	October/November 2023–2024; February/March 2023–2025; June/July 2023–2025

* NPs: natural products.

## Data Availability

Not applicable.
